# Positioning the Health Services Research Workforce for Continued Success: Recommendations from AcademyHealth Stakeholders

**DOI:** 10.1111/1475-6773.13039

**Published:** 2018-09-21

**Authors:** Nir Menachemi, Lisa A. Simpson, Meghan J. Wolfe

**Affiliations:** ^1^ Department of Health Policy and Management Richard M. Fairbanks School of Public Health Indiana University Indianapolis IN; ^2^ AcademyHealth Washington DC; ^3^ Department of Health Policy and Management Milken Institute School of Public Health The George Washington University Washington DC

**Keywords:** funding, training programs, workforce

## Abstract

AcademyHealth established a Workforce Initiative Task Force in 2016 to conduct an assessment of the state of the health services research workforce and develop recommendations for its future in the context of the changing health care and research ecosystems. This assessment included four components: a series of commissioned papers, an online priority setting process, a multistakeholder summit, and final analysis by the AcademyHealth Education Council. This paper presents this process and the resultant list of prioritized recommendations and planned next steps.

For at least fifty years, health services research (HSR) has played an important role in informing policies and practices to improve health and health care.^1^ Throughout the field's history, AcademyHealth, as the premier professional society for health services and policy research, along with its partners, has undertaken efforts to understand the HSR workforce, to inform the changing needs of the employers of researchers, and to guide the education of researchers to produce relevant evidence that informs decision making in the public and private sectors (Institute of Medicine 1979; Pittman and Holve 2009). In 2008, AcademyHealth first convened stakeholders in the HSR workforce, including training program directors and employers, as part of the HSR Learning Consortium. Stemming from this effort, in 2014, AcademyHealth established the Education Council^2^ to continue to focus on the HSR workforce and prepare it for the changing health policy and health systems context that have evolved following the passage of the Affordable Care Act. As its first priority, the Council recognized the need to periodically reassess the status of the HSR workforce, identify the future need for health services researchers, and to create an action plan for a vibrant HSR workforce.

AcademyHealth then established a Workforce Initiative Task Force with representatives of the Education Council and key funders^3^ to guide a systematic effort to understand the current health services research (HSR) workforce and plan for its future in the context of our changing health care and research ecosystems. The primary goals of this initiative were to (1) assess the current HSR workforce, (2) identify future demand for health services research, (3) create an action plan for the education of health services researchers, including recommendations for lifelong learning, and (4) actively disseminate the strategy to all relevant stakeholders. In recognition of the global nature of the issues at hand, AcademyHealth also partnered with colleagues at the Canadian Institutes for Health Research and at the World Health Organization's Alliance for Health Policy and Systems Research to include international dimensions to the activities. Papers were commissioned to inform the deliberations at a multistakeholder, invitational summit by assessing the current stock of health services researchers, the current and future demand for health services research, the training of health services researchers, and the core competencies for health services research.

Summit invitees were selected to represent the broad range of health service research stakeholders including directors of training programs, researchers at various career stages and in diverse employment settings, funders of health services research, journal editors, and users of health services research including health systems and policy professionals (characteristics of participants appear in Table 1). Representatives of the AcademyHealth Board of Directors, its three Councils (Education, Methods, and Corporate), its Membership Committee and student leadership were also included. While intended to be generally representative of the diverse voices and perspectives of the AcademyHealth membership and the broader health services research community, this was by no means a systematic sample. In advance of the summit, participants were expected to review draft versions of the commissioned papers to inform the summit deliberations and the priority setting process at the summit. Thus, the participants served as experts providing suggestions and recommendations inspired (in part) by the commissioned papers.

**Table 1:** Characteristics of Workforce Initiative Task Force Members Who Attended the Conference, October 2016

Variable	*N* (%)
HSR stakeholder type^a^	
Educator	15 (23.4)
Researcher	42 (65.6)
Employer	14 (21.9)
Funder	10 (15.6)
Student/Trainee	1 (1.6)
Publisher/Disseminator	3 (4.7)
Educational training of participants^a^	
PhD or similar	33 (51.6)
MD	18 (28.1)
JD	3 (4.7)
RN	4 (6.3)
Master's only	11 (17.2)
Total	64 (100%)

a Categories are not mutually exclusive; thus, percentages may not add up to 100%.

The summit was held in Washington, D.C. in October of 2016 and used a combination of plenary sessions where the commissioned papers were discussed and small workgroup sessions on how to best position the health services research workforce for continued future success. The workgroups tackled five aspects of the health services research workforce and identified recommendations for each. These five focus areas were supporting the training of health services researchers—programs, methods, and data; health services research core competencies; building a culture of health; improving the size and composition of the field of health services research; supporting health services research careers in the private sector; and maximizing the impact of health services research—dissemination and implementation.

After the summit, the recommendations were loaded onto a cloud based tool, Codigital, to support an online, consensus building activity to refine and validate the five sets of recommendations created by the workgroups. Codigital uses a pair wise voting process to refine and elevate which suggestions should “survive” through rounds of participant voting. For each set of recommendations, the list generated at the summit was available for review and modification for 1 week. Summit participants could suggest additional ideas or recommendations for the group to consider under each dimension, vote on the ideas/recommendations suggested during the summit, and make revisions to the ways the ideas/recommendations were articulated. The ultimate step in the development of the final list of recommendations presented in the current article was voting by the AcademyHealth Education Council. After review and discussion by the Council during a conference call, Council members were asked in an email survey to select their top three most important items, and separately, which items should AcademyHealth immediately begin addressing. The ordered list of prioritized action items based on this voting are displayed in Table 2. In sum, the ensuing recommendations should be regarded as informed commentary from a select group of experts and could be quite different than one might obtain from a probability sample of health services researchers providing feedback using a more deliberate scientific process.

**Table 2:** Prioritization of Action Items by AcademyHealth's Education Council

Rank Order Priority	Prioritized Specific Action Items	Who Should Be Responsible for Implementing?
1	Rather than conducting periodic enumerations of the HSR workforce, develop and confirm metrics of interest to measure changes in the HSR workforce and design a system to allow for the routine monitoring of the HSR workforce in relation to size, composition, and other relevant characteristics	AcademyHealth
2	Develop a handful of diverse but illustrative “career trajectories” of health services and policy researchers and use these for promotional purposes, particularly with respect to promoting HSR careers to high school students, undergraduate, and graduate students	AcademyHealth
3	Promote interdisciplinary learning experiences that integrate social determinants of health, population health, health policy, and dissemination of evidence‐based health information leading to informed health policy	AcademyHealth
4	Ensure that HSR curricula include the core concept of designing for dissemination and implementation, as well as key elements of implementation science	AcademyHealth
5	Provide students and trainees with access to a variety of experiential internship/fellowship opportunities to gain broader exposure to different areas of health care (e.g., delivery system, life‐sciences, research consulting organizations, data analytics companies, public health, other state or federal agencies.)	Individual HSR training programs
6	Population health and the social determinants of health should be added as a core competency in HSR training with opportunities for specialization: The curriculum should include experiential and community‐based training	Individual HSR training programs
7	Use Coursera or similar MOOC (massive open online course) to develop a foundational education resource for HSR programs focused on innovations in data science and data analytics that can be used broadly across many training programs/sites	Individual HSR training programs
8	Expand methods and analytics approaches by reaching beyond traditional HSR disciplines through collaborations with other fields (e.g., engineering, computer science, communications, and education)	Individual HSR training programs and/or journals that publish HSR
9	Help scientific journals evolve to be more proactive with dissemination. Articles should be written to emphasize application to health programs and policies and should include summaries written for lay readers and policymakers. Journals, authors, and funders should be trained to collaborate to augment dissemination	Journals that publish HSR
10	Share information about new methods, especially analyzing observational data in novel data sources, poll people to identify new/emerging methods. Understand new data sources and the challenges each bring	Individual HSR training programs and/or journals that publish HSR

## List of Recommendations

In all, 89 recommendations were generated by the five workgroups at the conference: 26 for improving the size and composition of the field; 16 for supporting the training of health services researchers; 20 for supporting careers in the private sector; 14 for maximizing the impact of health services research; and 13 for establishing a culture of health. These 89 recommendations included many similar suggestions and overarching themes. As such, two of the authors (MW and LS) synthesized the recommendations into ten distinct items, categorized into three action areas as follows:

### Action Area #1: Ensure a Diverse Workforce to Answer Priority Questions about Health and Health Care

#### Expand Awareness of Career Options in HSR

Making students aware of the variety of diverse career opportunities within HSR is essential to maintaining and expanding the size and scope of the workforce. Creating promotional materials on a range of HSR career trajectories was identified as a way to reach high school, undergraduate, and even graduate (particularly Masters) students who are likely unaware of the diverse career opportunities that training in HSR can provide. Conference participants expressed an interest in increasing partnership and recruitment activities with minority‐serving institutions and related associations to further diversify the pipeline. They also discussed the need for scholarships and other funding mechanisms to support students pursuing master degrees in HSR‐related fields.

#### Support Learning across Disciplines

Health services research is an inherently multidisciplinary field whose members are trained in a variety of disciplines spanning various clinical, public health, and social science specialties. Continued collaboration with related fields, disciplines, and stakeholder groups should be a priority for HSR training programs and the field at large in order to diversify the skills of health services researchers and facilitate innovative partnerships with other fields or sectors. Conference participants discussed supporting the development of dual degree programs with HSR doctoral degrees and related degree programs (e.g., JD, MD, MBA, EdD, MPH.) or with other disciplines (e.g., data science, communications.). They also suggested expanding methods and analytic approaches by moving beyond HSR and learning from other disciplines such as engineering, design, or data science.

#### Enhance Training with More Experiential Learning

One of the broadest areas of consensus that emerged from the conference was the need for students and trainees to engage in experiential learning during their education. This would allow students to gain broader exposure to different areas of HSR and sectors where HSR is produced and used, while providing them with skills that are increasingly important in the competitive employment marketplace. As part of this experience, a focus on acquiring essential soft skills was emphasized, including team membership, team leadership, management, and communication skills. Internships, fellowships, or other training programs in healthcare delivery systems, the life sciences industry, research consulting organizations, data analytics companies, or other state or federal agencies give students the opportunity to explore new and emerging sectors where HSR is produced and used, and also expands the reach of HSR in these spaces.

#### Support Life‐Long Learning of Health Services Researchers

While a focus on interdisciplinary training and experiential learning is essential to ensuring a high quality and diverse workforce, priority must also be given to support the continuing education of health services researchers, particularly in light of the rapid changes we are experiencing in data sources, technology, computing power, and analytics. Mentorship programs, especially in the private sector, and management and leadership training for mid‐level researchers leads to career advancement and prepares the next generation of senior leaders for mentorship of new HSR trainees. Additionally, programs in training departments aimed at faculty member rotations, adjunct positions, and sabbaticals through different organizations in industry and nonacademic settings provide learning opportunities for mid‐ and senior‐level researchers to expand their skill sets and gain new experiences. AcademyHealth should support these activities and develop programing and incentives for the continuing education of health services researchers across sectors.

### Action Area #2: Maximize the Impact of Health Services Research on Policy and Practice

#### Support the Diffusion of Data and Methods

Data and methods are foundational to producing rigorous and relevant evidence that can impact policy and practice. Sharing information about new methods (e.g., causal inference, predictive analytics, machine learning, qualitative techniques.) and data sources (EHR & HIE data, data from social service sectors, other big data sources, etc.) will allow health services researchers to utilize these new tools to frame research questions and study complex issues in novel and interesting ways. The field should encourage integrating these new and emerging data sources with more traditional data sources in a complementary way so that they are building on existing knowledge and resources to reach more robust and generalizable conclusions. AcademyHealth should develop online learning and virtual meetings to share innovations in data sources and new methods to broad audiences in the field.

#### Broaden the HSR Dissemination Skill Set

While peer‐reviewed journals will always be an important tool for dissemination of new HSR knowledge, it is clear that many other approaches to dissemination need to be adopted by researchers. Too often, training in alternative dissemination venues is not part of traditional HSR training and in some instances is in fact discouraged by academic programs. While it is clear that not every researcher will be skilled at communicating and translating their findings to a diverse set of audiences, they should understand those approaches and know how to seek out and partner with individuals and organizations who are. AcademyHealth should prioritize training in translation and communication with standardized and transparent use of terms and an emphasis on stakeholder engagement (see below). Fostering collaboration across disciplines who do dissemination well and teaching unique dissemination skills could strengthen the HSR dissemination skill set.

#### Support Dissemination as an Integral Part of Research Conduct

Though it is now widely accepted that dissemination is a fundamental part of the research process, funders do not always financially support these activities and the ability to accurately measure the impact of research dissemination efforts is in its infancy. AcademyHealth should recognize and promote funders who support grant‐making programs for integrated knowledge translation research. Metrics for measuring impact should be developed and standardized to assist researchers, funders, and stakeholders and included in the final grant reports, publications, and other research products. Journals should facilitate the training of editors and authors to be more proactive about dissemination by writing for the application of the research to policy and practice with stakeholder audiences in mind.

#### Involve Stakeholders throughout the Research Lifecycle

Attention to the involvement of stakeholders in the development, funding, research, and dissemination of research has grown in recent years, thanks in large part to the emphasis placed on this by PCORI. Some evidence exists that this engagement can result in more relevant evidence and ultimately use in action. By developing and promoting best practices for stakeholder engagement, the field can more readily address and respond to their needs and priorities. Several tactics could be implemented to address issues of stakeholder engagement, including training students in diverse settings so that they can be informed by stakeholder needs and getting stakeholders more involved in the academic process (e.g., dissertation/thesis committees, internships.).

### Action Area #3: Monitor the Health Services Workforce and Changes Over Time

#### Inform the Field's Efforts to Assess Its Effectiveness

A more continuous approach is needed to routinely monitor the size, composition, and other relevant characteristics of the HSR workforce. A guide outlining best practices for building the HSR workforce and a data repository for HSR training could aid this process. Creating metrics for measuring mentorship and the benefits of this type of relationship and encouraging faculty and senior leaders to mentor underrepresented minorities in the field are other ways to monitor and assess progress and changes in the workforce.

#### Identify New and Emerging Areas of HSR

To stay relevant and useful, the multidisciplinary nature of HSR requires periodic review and addition of new and emerging areas of practice, new methods and designs, as well as new types of data. The updated HSR doctoral core competencies reflect many of these changes, such as the inclusion of population health and the social determinants of health, health policy, and translation and dissemination in the core HSR training curriculum. The field must continue to promote and support multidisciplinary learning experiences in academia, the private sector, and other research agencies and organizations.

## Prioritizing the List

The highest ranked items, listed first in Table 2, correspond with the ninth recommendation above (*inform the field's efforts to access its effectiveness*). The second highest rank action item corresponds with the first recommendation (*expand awareness of career options in HSR*), and the third corresponds with the tenth (*identify new and emerging areas of HSR*).

Given discussion with the Education Council leadership and membership, the group decided to focus on the top four items on the prioritized list. Importantly, doing so was deemed consistent with AcademyHealth's role as an information broker for the field with responsibility for expanding awareness of HSR. The Education Council recommended that other remaining items on the prioritized list be implemented by individual HSR training programs and/or journals that publish HSR.

Over the next 18 months, AcademyHealth will work with its members and the Education Council to disseminate these recommendations and implement the four prioritized action items. As the field grows and changes, AcademyHealth will increase its role in monitoring the makeup of the HSR workforce and how it changes over time.

## Supporting information

Appendix SA1: Author Matrix.

## Notes

1 AcademyHealth defines *health services research* as the multidisciplinary field of scientific investigation that studies how social factors, financing systems, organizational structures and processes, health technologies, and personal behaviors affect access to health care, the quality and cost of health care, and ultimately our health and well‐being. Its research domains are individuals, families, organizations, institutions, communities, and populations.

2 AcademyHealth's Education Council members: http://www.academyhealth.org/about/people/councils/education-council.

3 Funders include the Agency for Healthcare Research and Quality (AHRQ), the Patient‐Centered Outcomes Research Institute (PCORI), and the Robert Wood Johnson Foundation (RWJF).
